# Resistance mechanisms in BRAF^V600E^ paediatric high-grade glioma and current therapeutic approaches

**DOI:** 10.3389/fonc.2022.1031378

**Published:** 2022-12-13

**Authors:** R. Lehmann, B. S. Rayner, D. S. Ziegler

**Affiliations:** ^1^ Children’s Cancer Institute, Lowy Cancer Research Centre, University of New South Wales (UNSW) Sydney, Sydney, NSW, Australia; ^2^ School of Clinical Medicine, University of New South Wales (UNSW) Medicine & Health, University of New South Wales (UNSW) Sydney, Sydney, NSW, Australia; ^3^ Kids Cancer Centre, Sydney Children’s Hospital, Randwick, NSW, Australia

**Keywords:** high grade glioma (HGG), resistance, BRAF^V600E^, MEK = mitogen extracellular kinase, pediatric

## Abstract

Paediatric high-grade gliomas (pHGG) are aggressive central nervous system tumours with a poor prognosis. BRAF^V600E^ mutant pHGGs can be treated with targeted BRAF inhibitors, which have shown both preclinical activity and potent clinical efficacy. Unfortunately, the development of drug resistance results in disease relapse or progression and is the primary cause of treatment failure. While there is a lot of data to explain mechanisms of resistance in other BRAF^V600E^ tumours, comparatively little is known about the mechanisms of BRAF inhibitor resistance in BRAF^V600E^ pHGG. Recent literature has identified aberrations in members of the RAS/RAF/ERK pathway, the PI3K/AKT/MTOR pathway and the cell cycle as major contributors to the resistance profile. A range of novel therapies have been suggested to overcome BRAF inhibitor drug resistance in BRAF^V600E^ pHGG. This review will discuss the current literature available for BRAF inhibitor resistant BRAF^V600E^ pHGGs and provide an overview of the currently available and proposed therapies.

## BRAF^V600E^ paediatric high-grade gliomas

Paediatric high-grade gliomas (pHGG) are a subset of central nervous system (CNS) tumours with a poor prognosis and low survival (1–4). For many pHGG subtypes there are few treatments available, with palliative treatments the only option for many. BRAF^V600E^ mutations are seen in both paediatric low-grade glioma (pLGG) and pHGG, with an overall prevalence in pHGG of approximately 6% across the tumour subtypes (5–9). In pLGG, prognosis is poorer for patients with BRAF^V600E^ mutant tumours, and these tumours have a higher likelihood of transforming to HGGs (10), Indeed, BRAF^V600E^ is the most common recurrent mutation in these secondary pHGG, occurring in 39% of cases (11). Interestingly, in pHGG BRAF^V600E^ mutations confer an improved prognosis compared to wildtype tumours, with a 2 year survival of 67% (5). However, secondary mutations such as *CDKN2A/B* deletion or *TERT* promoter mutations, frequently co-occur with BRAF^V600E^, with evidence demonstrating that these mutations lead to more aggressive tumours and a poorer prognosis (8, 12, 13). While most BRAF^V600E^ mutant pHGG respond well to BRAF inhibition, the response is confounded by the rapid onset of drug resistance. Until recently, limited research has been performed on drug resistance in BRAF^V600E^ pHGG, with most studies focussing on pLGG and adult HGG cell lines studies in place of pHGG (14, 15).

The therapeutic use of targeted BRAF inhibitors have shown improved survival in adults with BRAF^V600E^ mutated melanoma in Phase 3 clinical trials and are now part of standard clinical care, with first generation BRAF inhibitors vemurafenib and dabrafenib approved by the FDA in 2011 and 2013 respectively (16, 17). This has informed several paediatric tumour studies (10, 18–20), with BRAF inhibitors since showing promise in pHGG (21, 22). Vemurafenib is currently being investigated through the Children’s Oncology Group MATCH trial for its efficacy in treating paediatric tumours harbouring BRAF^V600^ mutations, including pHGG (NCT03155620, NCT03220035). Unfortunately, the onset of resistance to first line BRAF inhibitors is a common outcome, thought to be due to reactivation of the MEK pathway, as occurs in other tumours (23). One strategy to mitigate against this phenomenon is to combine BRAF inhibitors with MEK inhibitors, which is being investigated in several ongoing clinical trials (NCT03919071, NCT04201457, NCT02684058) in BRAF^V600E^ pHGG (24). Recent findings from one of these phase II clinical trials (NCT02684058) reported that the combination of dabrafenib and trametinib treatment led to a 56% response rate and 66% clinical benefit rate in children with recurrent or refractory pHGG (25). However, the majority of patients demonstrated tumour progression within 12 months, indicating that the development of resistance also occurs following treatment with combination BRAF/MEK inhibitor therapy, as seen in other tumour types (26, 27).

## BRAF^V600E^ pHGG resistance mechanisms

**Figure 1:**
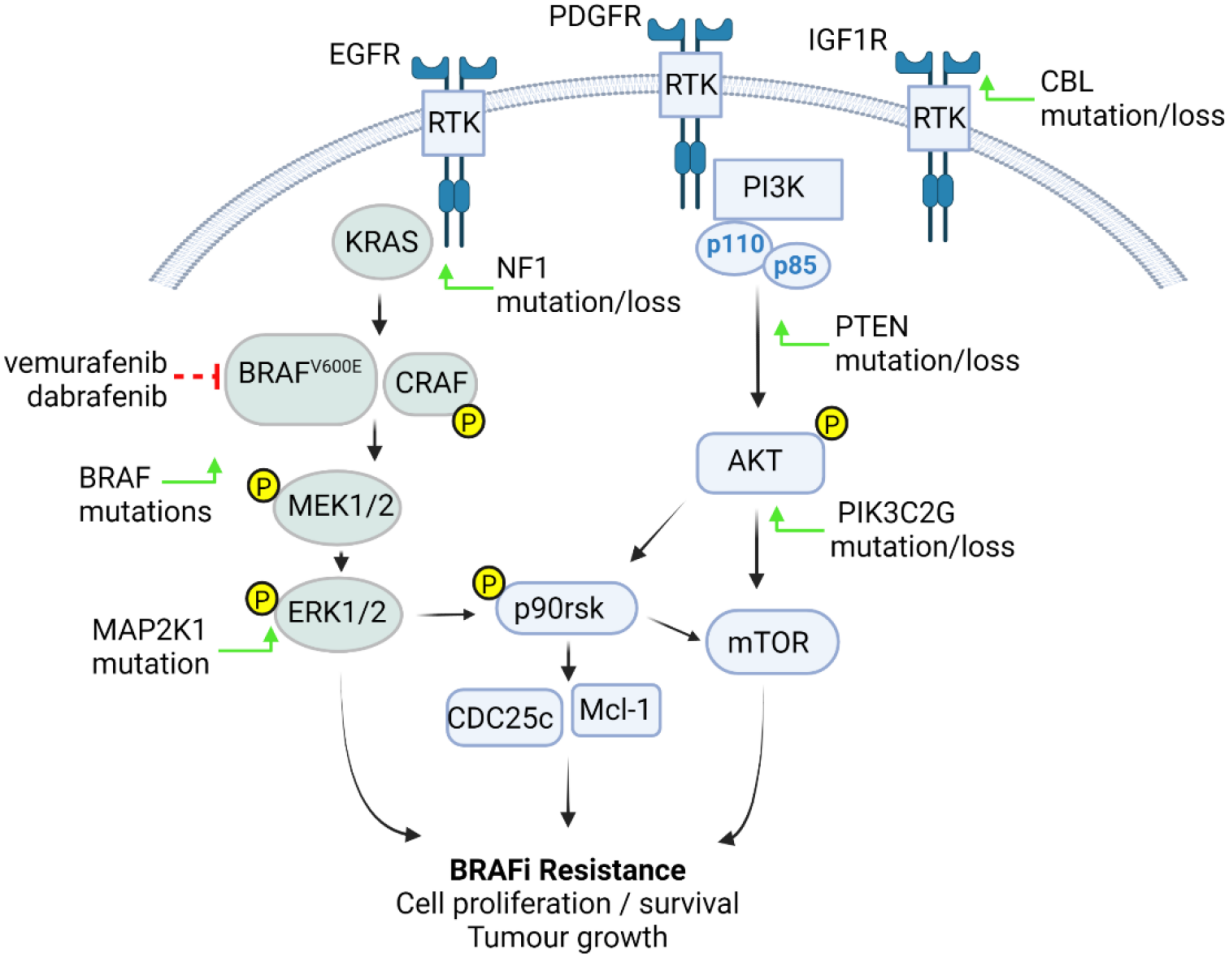
BRAF^V600E^ pHGG resistance mechanisms. Tumour cells become resistant to BRAFi through increased activation of the RAS-RAF- MEK-ERK or PI3K-AKT pathways. These events are driven by upregulation or activation of receptor tyrosine kinases (RTK) such as EGFR, PDGFRβ and IGF1R through either BRAF mutations or CBL, NF1, PTEN, MAP2K1, or PIK3C2G mutation or loss (green arrows), resulting in pathway phosphorylation and activation of downstream p90rsk, Mcl-1 and cell cycle components, leading increased cell proliferation, survival and tumour growth (figure created with BioRender.com).

The most commonly identified mechanisms of resistance in BRAF^V600E^ mutated tumours are due to reactivation of the RAS/RAF/ERK pathway (**Figure 1**). Much of the research into resistance mechanisms has been conducted in melanoma, due to the high prevalence of the BRAF^V600E^ mutation in this disease (28). Common underlying mechanisms include upregulation of receptor tyrosine kinases (RTKs): EGFR, PDGF-β, and IGF1R, mutational activation of KRAS and NRAS, aberrant BRAF splicing, RAF isoform switching, BRAF^V600E^ dimerisation and BRAF^V600E^ amplification (29–34). Additionally, upregulated Wnt signalling, *Mcl*-1 overexpression (35, 36), as well as Sterol Regulatory Element-Binding Protein (SREBP1) activation and subsequent lipogenesis have been implicated in BRAF inhibitor resistance (37, 38).

In contrast to melanoma, there has been less investigation into the mechanisms of resistance in BRAF^V600E^ pHGG. Consistent with the findings in melanoma, however, reactivation of the RAS/RAF/ERK has been implicated in resistance to BRAF inhibitors in BRAF^V600E^ pHGG. Further, a secondary point mutation has been identified as a driver of resistance in a case study of BRAF^V600E^ paediatric glioma (39). Through whole-exome sequencing Wang et al., identified a secondary *BRAF* ^L514V^ mutation, which was present following tumour progression, but not in the pre-dabrafenib treatment tumour. This mutation directly induced ERK signalling, promoted RAF dimer formation, and was sufficient to confer resistance to dabrafenib (39). Similarly, next generation sequencing has been used on both pre- and post-BRAF inhibitor treated BRAF^V600E^ HGG and LGG from paediatric and young adult patients to identify novel mutations that act as putative drivers of resistance (13). These identified mutations in genes that modulate RTK activity, such as *CBL*, alterations in RAS/RAF signalling through *NF1* missense mutations, secondary point mutations of *BRAF*, activating mutations of *MAP2K1* and mutations in *PTEN*. Alterations in the PI3K/AKT/mTOR pathway have also been identified as a mechanism of resistance in pHGG, with mutations in *PIK3C2G* and upregulation of p-AKT identified (13, 40). Furthermore, mutations in *BAP* and *ANKHD1* in post-BRAF inhibitor treated tissues have also implicated cell cycle drivers as contributors towards BRAF inhibitor resistance (13). Further validation of *NF1, CBL* and *PTEN* loss, performed in several pLGG and adult HGG BRAF^V600E^ cell lines (but not pHGG), confirmed their roles as drivers of BRAF inhibitor resistance. CRAF-dominated signalling and alterations in KRAS and EGFR signalling were also identified as drivers of resistance. Subsequent mechanistic analyses for vemurafenib resistance were undertaken using drug resistant lines developed from BRAF^V600E^-mutant glioma cell lines AM38 (BRAF^V600E^ adult HGG) and MAF-794 (BRAF^V600E^ pLGG). Single-nucleotide variants conferring functional alterations in *ERRFI1* ^S251*^ and *TET2* ^V1199E^ were observed in each of the respective resistant cell lines, suggestive of possible mechanisms of BRAF inhibitor resistance *in vitro* (13). Baseline upregulation of several RTKs, RAS, p-CRAF and p-p90rsk were also seen in three BRAF^V600E^ HGG tumours (including one paediatric and one adolescent sample) collected following relapse after BRAF inhibitor treatment (40). Interestingly, in contrast to Schreck et al. (13), no known mutations of RAS, MAPK or PI3K/AKT were identified in the resistant tumour samples and no novel driver mutations were able to be identified (40). Additionally, DNA methylation status was also largely unaffected in these patients following BRAF inhibition treatment, overall indicating that nongenomic and non-epigenomic events may also be involved in driving resistance mechanisms (40).

## Treatments to overcome resistance

As discussed above, a common strategy used to overcome resistance to BRAF inhibitors is to use combination treatments (41). With MEK reactivation being the most common mechanism of drug resistance in BRAF*^V600E^* tumours, MEK inhibitors are a frequently used combination therapy, with several MEK inhibitors approved for clinical use. Dual BRAF and MEK inhibition has demonstrated promising results in several other BRAF^V600E^ tumour types (42–44). The efficacy of these treatment regimens are now being tested within the setting of pHGG. Pre-clinical studies in a BRAF^V600E^ expressing Ink4a-Arf knock-out mouse HGG model showed that dabrafenib monotherapy had no significant impact on survival. Further, *ex vivo* dabrafenib treatment of HGG cells derived from these mice resulted in activation of EGFR and AKT, that was concurrent with p-ERK increases (45). However, it should be noted that this was a model of intrinsic, rather than acquired resistance. Regardless, subsequent combination treatment with dabrafenib and the MEK inhibitor, trametinib, significantly improved survival and reduced Ki67 staining compared to the control group (45), mirroring the aforementioned preliminary phase II clinical trial findings (24, 25).

Despite these promising early results, it is well established in other tumour types that resistance to this combination therapy frequently develops (26, 27). In support of this, other combination therapies targeting multiple, rather than single pathways, have been trialled with some success in BRAF^V600E^ pHGG preclinical models. For example, HSP90 is a chaperone protein, known to associate with RTKs and several proteins shared between the MAPK and AKT/mTOR pathways (46–49). In support of a potential role of this chaperone in resistance mechanisms, HSP90 inhibitors have been found to have a synergistic response in combination with either dabrafenib or trametinib in both primary HGGs, as well as in HGGs collected following relapse after BRAF inhibitor treatment. While these treatments did not completely negate the tumorgenicity of the resistant HGG phenotype, the combination therapies did significantly improve the response when compared to single agent treatments. Importantly, the addition of an HSP90 inhibitor, in combination with dabrafenib or trametinib was also effective in two BRAF^V600E^ HGG BRAF/MEK inhibitor resistant PDX models, with tumour regression most evident in the paediatric model (40).

A range of novel inhibitors have been developed that may overcome drug resistance in BRAF^V600E^ pHGG. Targeted first generation BRAF inhibitors act upon BRAF monomers, preventing their dimerisation. As a resistance mechanism, reactivation of the RAS/RAF/ERK pathway frequently occurs through secondary mutations which promote RAF dimerisation, independent of RAS activation (29, 50). Therefore, dimer disrupters and pan-RAF inhibitors, which act upon RAF dimers as opposed to BRAF monomers, are being explored as novel therapies. Dimer disrupters prevent dimer formation, resulting in inhibition of downstream ERK signalling (51). Dimer disrupters have shown efficacy against BRAF^V600E^ dimer forming melanoma and colorectal cancer cells and PDX models (51) and are currently in clinical trial for both paediatric and adult activating BRAF V600E and non-V600E mutant tumours, including brain tumours (NCT02428712). Pan-Raf inhibitors act by binding equally to both of the RAF monomers that form the RAF dimer, and therefore can inhibit RAF signalling in BRAF^V600E^ tumours which exhibit RAF dimerisation (52). Pan-Raf inhibitors have been shown to display efficacy within the CNS and demonstrate blood brain barrier permeability properties (53, 54).The pan-RAF inhibitors, LY3009120 and belvarafenib, were efficacious in an intrinsically RAF inhibitor resistant glioma line. These cells were isolated from a tumour removed from a patient at the Children’s Hospital Colorado and found to be resistant to BRAF inhibitors (13, 55). Interestingly, despite these cells demonstrating resistance to the BRAF inhibitor vemurafenib, combining the pan-RAF inhibitor LY3009120 with vemurafenib resulted in enhanced cytotoxicity, over LY3009120 treatment alone (13). These studies highlight the potential of combining pan-RAF and MEK inhibitor therapy in order to overcome drug resistance in pHGG and are supported by such treatment regimens displaying efficacy in drug resistant melanoma and colorectal cancer (56, 57).

Indeed, the identification of new pathways to target in combination with BRAF inhibition may be more effective than targeting a single pathway. Next generation sequencing is likely to contribute significantly towards identifying these targets. For example, inhibition of cellular autophagy pathways has already been identified as an area of interest for drug resistant BRAF^V600E^ pHGG. Autophagy inhibition has been shown to reverse drug resistance in BRAF^V600E^ glioma, with combined chloroquine and vemurafenib treatment reducing cell growth in vemurafenib resistant adult and paediatric glioma cell lines and cultures (58). A phase I/II trial (NCT04201457) is currently underway to ascertain the effectiveness of this approach, using hydroxychloroquine in combination with the BRAF inhibitor dabrafenib and/or the MEK inhibitor trametinib in BRAF^V600E^ pHGG.

## The future for BRAF^V600E^ pHGG research

Promising results are beginning to emerge on overcoming drug resistance in BRAF^V600E^ pHGG. However, future preclinical studies will need to have a greater focus on pHGG models to help elucidate the biology of this disease. To date there have been no reports using tissues and/or cells of pHGG origin to study the mechanisms of resistance, with both adult HGG or pLGG samples or cells lines being substituted. Due to distinct differences, both genomic and phenotypic, between HGG and LGG, as well as between paediatric and adult tumours, the development of specific pHGG models will help direct clinical trials and future treatment strategies.

## Conclusions

BRAF^V600E^ pHGG is a rare CNS tumour with a poor prognosis, which responds well to targeted therapy; however, treatment is limited by the rapid onset of resistance. Whilst significant progress has been made in determining the underlying mechanisms of resistance in other BRAF^V600E^ cancers, drug resistance in BRAF^V600E^ pHGG is less well understood. Recent studies have helped to begin to elucidate these mechanisms. Already, the identification of novel therapies such as dimer disrupters and pan-RAF inhibitors are an exciting development for the field. It is hoped that further identification of resistance mechanisms in BRAF^V600E^ pHGG will facilitate further improvements in patient outcomes.

## Author contributions

RL wrote the first draft of this manuscript and reviewed subsequent iterations, BR and DZ wrote and reviewed this manuscript. All authors contributed to the article and approved the submitted version.

## References

[1] Napieralska A Krzywon A Mizia-Malarz A Sosna-Zielińska J Pawłowska E Krawczyk MA . High-grade gliomas in children-a multi-institutional polish study. Cancers (Basel) (2021) 13(9):2062. doi: 10.3390/cancers13092062 33923337 PMC8123180

[2] Broniscer A Gajjar A . Supratentorial high-grade astrocytoma and diffuse brainstem glioma: two challenges for the pediatric oncologist. Oncologist (2004) 9(2):197–206. doi: 10.1634/theoncologist.9-2-197 15047924

[3] Finlay JL Boyett JM Yates AJ Wisoff JH Milstein JM Geyer JR . Randomized phase III trial in childhood high-grade astrocytoma comparing vincristine, lomustine, and prednisone with the eight-drugs-in-1-day regimen. childrens cancer group. J Clin Oncol (1995) 13(1):112–23. doi: 10.1200/JCO.1995.13.1.112 7799011

[4] Cohen KJ Pollack IF Zhou T Buxton A Holmes EJ Burger PC . Temozolomide in the treatment of high-grade gliomas in children: a report from the children's oncology group. Neuro-Oncology (2011) 13(3):317–23. doi: 10.1093/neuonc/noq191 PMC3064602 21339192

[5] Mackay A Burford A Carvalho D Izquierdo E Fazal-Salom J Taylor KR . Integrated molecular meta-analysis of 1,000 pediatric high-grade and diffuse intrinsic pontine glioma. Cancer Cell (2017) 32(4):520–537.e5. doi: 10.1016/j.ccell.2017.08.017 28966033 PMC5637314

[6] Nicolaides TP Li H Solomon DA Hariono S Hashizume R Barkovich K . Targeted therapy for BRAFV600E malignant astrocytoma. Clin Cancer Res (2011) 17(24):7595–604. doi: 10.1158/1078-0432.CCR-11-1456 PMC3638050 22038996

[7] Guidi M Giunti L Buccoliero AM Caporalini C Censullo ML Galli L . Genetic signature and treatment of pediatric high-grade glioma. Mol Clin Oncol (2021) 14(4):70. doi: 10.3892/mco.2021.2232 33732456 PMC7907799

[8] Schiffman JD Hodgson JG VandenBerg SR Flaherty P Polley MY Yu M . Oncogenic BRAF mutation with CDKN2A inactivation is characteristic of a subset of pediatric malignant astrocytomas. Cancer Res (2010) 70(2):512–9. doi: 10.1158/0008-5472.CAN-09-1851 PMC2851233 20068183

[9] Ryall S Tabori U Hawkins C . Pediatric low-grade glioma in the era of molecular diagnostics. Acta Neuropathologica Commun (2020) 8(1):30. doi: 10.1186/s40478-020-00902-z PMC7066826 32164789

[10] Nobre L Zapotocky M Ramaswamy V Ryall S Bennett J Alderete D . Outcomes of BRAF V600E pediatric gliomas treated with targeted BRAF inhibition. JCO Precis Oncol (2020) 4):561–71. doi: 10.1200/PO.19.00298 PMC7446502 32923898

[11] Mistry M Zhukova N Merico D Rakopoulos P Krishnatry R Shago M . BRAF mutation and CDKN2A deletion define a clinically distinct subgroup of childhood secondary high-grade glioma. J Clin Oncol (2015) 33(9):1015–22. doi: 10.1200/JCO.2014.58.3922 PMC4356711 25667294

[12] Gabler L Lötsch D Kirchhofer D van Schoonhoven S Schmidt HM Mayr L . TERT expression is susceptible to BRAF and ETS-factor inhibition in BRAF(V600E)/TERT promoter double-mutated glioma. Acta Neuropathol Commun (2019) 7(1):128. doi: 10.1186/s40478-019-0775-6 31391125 PMC6685154

[13] Schreck KC Morin A Zhao G Allen AN Flannery P Glantz M . Deconvoluting mechanisms of acquired resistance to RAF inhibitors in BRAF(V600E)-mutant human glioma. Clin Cancer Res (2021) 27(22):6197–208. doi: 10.1158/1078-0432.CCR-21-2660 PMC8595717 34433654

[14] Yao TW Zhang J Prados M Weiss WA James CD Nicolaides T . Acquired resistance to BRAF inhibition in BRAFV600E mutant gliomas. Oncotarget (2017) 8(1):583–95. doi: 10.18632/oncotarget.11882 PMC5352180 27611946

[15] Zhang J Yao TW Hashizume R Hariono S Barkovich KJ Fan QW . Combined BRAF(V600E) and MEK blockade for BRAF(V600E)-mutant gliomas. J Neurooncol (2017) 131(3):495–505. doi: 10.1007/s11060-016-2333-4 27848137 PMC5560871

[16] Sosman JA Kim KB Schuchter L Gonzalez R Pavlick AC Weber JS . Survival in BRAF V600–mutant advanced melanoma treated with vemurafenib. New Engl J Med (2012) 366(8):707–14. doi: 10.1056/NEJMoa1112302 PMC3724515 22356324

[17] Hauschild A Grob JJ Demidov LV Jouary T Gutzmer R Millward M . Dabrafenib in BRAF-mutated metastatic melanoma: a multicentre, open-label, phase 3 randomised controlled trial. Lancet (2012) 380(9839):358–65. doi: 10.1016/S0140-6736(12)60868-X 22735384

[18] Kieran MW Bouffet E Broniscer A Cohen KJ Geoerger B Hansford JR . Efficacy and safety results from a phase I/IIa study of dabrafenib in pediatric patients with BRAF V600–mutant relapsed refractory low-grade glioma. J Clin Oncol (2018) 36(15_suppl):10506–6. doi: 10.1200/JCO.2018.36.15_suppl.10506

[19] Nicolaides T Nazemi KJ Crawford J Kilburn L Minturn J Gajjar A . Phase I study of vemurafenib in children with recurrent or progressive BRAF(V600E) mutant brain tumors: Pacific pediatric neuro-oncology consortium study (PNOC-002). Oncotarget (2020) 11(21):1942–52. doi: 10.18632/oncotarget.27600 PMC7260122 32523649

[20] Chisholm JC Suvada J Dunkel IJ Casanova M Zhang W Ritchie N . BRIM-p: A phase I, open-label, multicenter, dose-escalation study of vemurafenib in pediatric patients with surgically incurable, BRAF mutation-positive melanoma. Pediatr Blood Cancer (2018) 65(5):e26947. doi: 10.1002/pbc.26947 29350463 PMC5867229

[21] Robinson GW Orr BA Gajjar A . Complete clinical regression of a BRAF V600E-mutant pediatric glioblastoma multiforme after BRAF inhibitor therapy. BMC Cancer (2014) 14(1):258. doi: 10.1186/1471-2407-14-258 24725538 PMC3996187

[22] Bautista F Paci A Minard-Colin V Dufour C Grill J Lacroix L . Vemurafenib in pediatric patients with BRAFV600E mutated high-grade gliomas. Pediatr Blood Cancer (2014) 61(6):1101–3. doi: 10.1002/pbc.24891 24375920

[23] Long GV Stroyakovskiy D Gogas H Levchenko E de Braud F Larkin J . Combined BRAF and MEK inhibition versus BRAF inhibition alone in melanoma. N Engl J Med (2014) 371(20):1877–88. doi: 10.1056/NEJMoa1406037 25265492

[24] Toll SA Tran HN Cotter J Judkins AR Tamrazi B Biegel JA . Sustained response of three pediatric BRAF(V600E) mutated high-grade gliomas to combined BRAF and MEK inhibitor therapy. Oncotarget (2019) 10(4):551–7. doi: 10.18632/oncotarget.26560 PMC6355184 30728904

[25] Hargrave DR Terashima K Hara J Kordes UR Upadhyaya SA Sahm F . Dabrafenib + trametinib (dab + tram) in relapsed/refractory (r/r) BRAF V600–mutant pediatric high-grade glioma (pHGG): Primary analysis of a phase II trial. J Clin Oncol (2022) 40(16_suppl):2009–9. doi: 10.1200/JCO.2022.40.16_suppl.2009 PMC10666989 37643378

[26] Tripathi R Liu Z Jain A Lyon A Meeks C Richards D . Combating acquired resistance to MAPK inhibitors in melanoma by targeting Abl1/2-mediated reactivation of MEK/ERK/MYC signaling. Nat Commun (2020) 11(1):5463. doi: 10.1038/s41467-020-19075-3 33122628 PMC7596241

[27] Wang B Zhang W Zhang G Kwong L Lu H Tan J . Targeting mTOR signaling overcomes acquired resistance to combined BRAF and MEK inhibition in BRAF-mutant melanoma. Oncogene (2021) 40(37):5590–9. doi: 10.1038/s41388-021-01911-5 PMC8445818 34304249

[28] Ascierto PA Kirkwood JM Grob JJ Simeone E Grimaldi AM Maio M . The role of BRAF V600 mutation in melanoma. J Transl Med (2012) 10:85. doi: 10.1186/1479-5876-10-85 22554099 PMC3391993

[29] Poulikakos PI Persaud Y Janakiraman M Kong X Ng C Moriceau G . RAF Inhibitor resistance is mediated by dimerization of aberrantly spliced BRAF(V600E). Nature (2011) 480(7377):387–90. doi: 10.1038/nature10662 PMC3266695 22113612

[30] Nazarian R Shi H Wang Q Kong X Koya RC Lee H . Melanomas acquire resistance to b-RAF(V600E) inhibition by RTK or n-RAS upregulation. Nature (2010) 468(7326):973–7. doi: 10.1038/nature09626 PMC3143360 21107323

[31] Villanueva J Vultur A Lee JT Somasundaram R Fukunaga-Kalabis M Cipolla AK . Acquired resistance to BRAF inhibitors mediated by a RAF kinase switch in melanoma can be overcome by cotargeting MEK and IGF-1R/PI3K. Cancer Cell (2010) 18(6):683–95. doi: 10.1016/j.ccr.2010.11.023 PMC3026446 21156289

[32] Dumaz N . Mechanism of RAF isoform switching induced by oncogenic RAS in melanoma. Small GTPases (2011) 2(5):289–92. doi: 10.4161/sgtp.2.5.17814 PMC3265821 22292133

[33] Su F Bradley WD Wang Q Yang H Xu L Higgins B . Resistance to selective BRAF inhibition can be mediated by modest upstream pathway activation. Cancer Res (2012) 72(4):969–78. doi: 10.1158/0008-5472.CAN-11-1875 22205714

[34] Shi H Moriceau G Kong X Lee MK Lee H Koya RC . Melanoma whole-exome sequencing identifies (V600E)B-RAF amplification-mediated acquired b-RAF inhibitor resistance. Nat Commun (2012) 3:724. doi: 10.1038/ncomms1727 22395615 PMC3530385

[35] Anastas JN Kulikauskas RM Tamir T Rizos H Long GV von Euw EM . WNT5A enhances resistance of melanoma cells to targeted BRAF inhibitors. J Clin Invest (2014) 124(7):2877–90. doi: 10.1172/JCI70156 PMC4071371 24865425

[36] Fofaria NM Frederick DT Sullivan RJ Flaherty KT Srivastava SK . Overexpression of mcl-1 confers resistance to BRAFV600E inhibitors alone and in combination with MEK1/2 inhibitors in melanoma. Oncotarget (2015) 6(38):40535–56. doi: 10.18632/oncotarget.5755 PMC4747351 26497853

[37] Crocco M Verrico A Milanaccio C Piccolo G De Marco P Gaggero G . Dyslipidemia in children treated with a BRAF inhibitor for low-grade gliomas: A new side effect? Cancers (Basel) (2022) 14(11):2693. doi: 10.3390/cancers14112693 35681673 PMC9179293

[38] Talebi A Dehairs J Rambow F Rogiers A Nittner D Derua R . Sustained SREBP-1-dependent lipogenesis as a key mediator of resistance to BRAF-targeted therapy. Nat Commun (2018) 9(1):2500. doi: 10.1038/s41467-018-04664-0 29950559 PMC6021375

[39] Wang J Yao Z Jonsson P Allen AN Qin ACR Uddin S . A secondary mutation in BRAF confers resistance to RAF inhibition in a BRAF(V600E)-mutant brain tumor. Cancer Discovery (2018) 8(9):1130–41. doi: 10.1158/2159-8290.CD-17-1263 PMC6125191 29880583

[40] Sasame J Ikegaya N Kawazu M Natsumeda M Hayashi T Isoda M . HSP90 inhibition overcomes resistance to molecular targeted therapy in BRAFV600E-mutant high-grade glioma. Clin Cancer Res (2022) 28(11):2425–39. doi: 10.1158/1078-0432.CCR-21-3622 35344043

[41] Proietti I Skroza N Bernardini N Tolino E Balduzzi V Marchesiello A . Mechanisms of acquired BRAF inhibitor resistance in melanoma: A systematic review. Cancers (Basel) (2020) 12(10):2801. doi: 10.3390/cancers12102801 33003483 PMC7600801

[42] Flaherty KT Infante JR Daud A Gonzalez R Kefford RF Sosman J . Combined BRAF and MEK inhibition in melanoma with BRAF V600 mutations. N Engl J Med (2012) 367(18):1694–703. doi: 10.1056/NEJMoa1210093 PMC3549295 23020132

[43] Planchard D Besse B Kim TM Quoix EA Souquet PJ Mazieres J . Updated survival of patients (pts) with previously treated BRAF V600E–mutant advanced non-small cell lung cancer (NSCLC) who received dabrafenib (D) or d + trametinib (T) in the phase II BRF113928 study. J Clin Oncol (2017) 35(15_suppl):9075–5. doi: 10.1200/JCO.2017.35.15_suppl.9075

[44] Ascierto PA McArthur GA Dréno B Atkinson V Liszkay G Di Giacomo AM . Cobimetinib combined with vemurafenib in advanced BRAF(V600)-mutant melanoma (coBRIM): updated efficacy results from a randomised, double-blind, phase 3 trial. Lancet Oncol (2016) 17(9):1248–60. doi: 10.1016/S1470-2045(16)30122-X 27480103

[45] Grossauer S Koeck K Murphy NE Meyers ID Daynac M Truffaux N . Concurrent MEK targeted therapy prevents MAPK pathway reactivation during BRAFV600E targeted inhibition in a novel syngeneic murine glioma model. Oncotarget (2016) 7(46):75839–53. doi: 10.18632/oncotarget.12419 PMC5342782 27713119

[46] Sreedhar AS Söti C Csermely P . Inhibition of Hsp90: a new strategy for inhibiting protein kinases. Biochim Biophys Acta (BBA) - Proteins Proteomics (2004) 1697(1):233–42. doi: 10.1016/j.bbapap.2003.11.027 15023364

[47] Schopf FH Biebl MM Buchner J . The HSP90 chaperone machinery. Nat Rev Mol Cell Biol (2017) 18(6):345–60. doi: 10.1038/nrm.2017.20 28429788

[48] Giulino-Roth L van Besien HJ Dalton T Totonchy JE Rodina A Taldone T . Inhibition of Hsp90 suppresses PI3K/AKT/mTOR signaling and has antitumor activity in burkitt lymphoma. Mol Cancer Ther (2017) 16(9):1779–90. doi: 10.1158/1535-7163.MCT-16-0848 PMC5587381 28619753

[49] Haupt A Joberty G Bantscheff M Fröhlich H Stehr H Schweiger MR . Hsp90 inhibition differentially destabilises MAP kinase and TGF-beta signalling components in cancer cells revealed by kinase-targeted chemoproteomics. BMC Cancer (2012) 12(1):38. doi: 10.1186/1471-2407-12-38 22277058 PMC3342885

[50] Yao Z Torres NM Tao A Gao Y Luo L Li Q . BRAF mutants evade ERK-dependent feedback by different mechanisms that determine their sensitivity to pharmacologic inhibition. Cancer Cell (2015) 28(3):370–83. doi: 10.1016/j.ccell.2015.08.001 PMC4894664 26343582

[51] Yao Z Gao Y Su W Yaeger R Tao J Na N . RAF Inhibitor PLX8394 selectively disrupts BRAF dimers and RAS-independent BRAF-mutant-driven signaling. Nat Med (2019) 25(2):284–91. doi: 10.1038/s41591-018-0274-5 PMC6404779 30559419

[52] Peng SB Henry JR Kaufman MD Lu WP Smith BD Vogeti S . Inhibition of RAF isoforms and active dimers by LY3009120 leads to anti-tumor activities in RAS or BRAF mutant cancers. Cancer Cell (2015) 28(3):384–98. doi: 10.1016/j.ccell.2015.08.002 26343583

[53] Gampa G Kim M Mohammad AS Parrish KE Mladek AC Sarkaria JN . Brain distribution and active efflux of three panRAF inhibitors: Considerations in the treatment of melanoma brain metastases. J Pharmacol Exp Ther (2019) 368(3):446. doi: 10.1124/jpet.118.253708 30622172 PMC6374543

[54] Sun Y Alberta JA Pilarz C Calligaris D Chadwick EJ Ramkissoon S . A brain-penetrant RAF dimer antagonist for the noncanonical BRAF oncoprotein of pediatric low-grade astrocytomas. Neuro Oncol (2017) 19(6):774–85. doi: 10.1093/neuonc/now261 PMC5464455 28082416

[55] Crespo M Zahedi S Morin A Wodetzki D Caino C Levy JM . LGG-55. autophagy sensitizes CNS tumors to targeted therapy by lowering their apoptotic threshold. Neuro-oncology (2022) 24(Suppl 1):i101–1. doi: 10.1093/neuonc/noac079.367

[56] Whittaker SR Cowley GS Wagner S Luo F Root DE Garraway LA . Combined pan-RAF and MEK inhibition overcomes multiple resistance mechanisms to selective RAF inhibitors. Mol Cancer Ther (2015) 14(12):2700–11. doi: 10.1158/1535-7163.MCT-15-0136-T PMC4674359 26351322

[57] Nakamura A Arita T Tsuchiya S Donelan J Chouitar J Carideo E . Antitumor activity of the selective pan-RAF inhibitor TAK-632 in BRAF inhibitor-resistant melanoma. Cancer Res (2013) 73(23):7043–55. doi: 10.1158/0008-5472.CAN-13-1825 24121489

[58] Mulcahy Levy JM Zahedi S Griesinger AM Morin A Davies KD Aisner DL . Autophagy inhibition overcomes multiple mechanisms of resistance to BRAF inhibition in brain tumors. Elife (2017) 6. doi: 10.7554/eLife.19671 PMC5241115 28094001

